# Quantitative analysis of the effect of environmental-scanning electron microscopy on collagenous tissues

**DOI:** 10.1038/s41598-018-26839-x

**Published:** 2018-05-31

**Authors:** Woowon Lee, Kimani C. Toussaint

**Affiliations:** 1 University of Illinois at Urbana-Champaign, Department of Mechanical Science and Engineering, 1206 W Green Street, Urbana, Illinois 61801 United States; 2 University of Illinois at Urbana-Champaign, PROBE Lab, 1206 W Green Street, Urbana, Illinois 61801 United States; 3 University of Illinois at Urbana-Champaign, Affiliate in the Department of Electrical and Computer Engineering, 1406 W Green Street, Urbana, Illinois 61801 United States; 4 University of Illinois at Urbana-Champaign, Department of Bioengineering, 1270 Digital Computer Laboratory, Urbana, Illinois 61801 United States

## Abstract

Environmental-scanning electron microscopy (ESEM) is routinely applied to various biological samples due to its ability to maintain a wet environment while imaging; moreover, the technique obviates the need for sample coating. However, there is limited research carried out on electron-beam (e-beam) induced tissue damage resulting from using the ESEM. In this paper, we use quantitative second-harmonic generation (SHG) microscopy to examine the effects of e-beam exposure from the ESEM on collagenous tissue samples prepared as either fixed, frozen, wet or dehydrated. Quantitative SHG analysis of tissues, before and after ESEM e-beam exposure in low-vacuum mode, reveals evidence of cross-linking of collagen fibers, however there are no structural differences observed in fixed tissue. Meanwhile wet-mode ESEM appears to radically alter the structure from a regular fibrous arrangement to a more random fiber orientation. We also confirm that ESEM images of collagenous tissues show higher spatial resolution compared to SHG microscopy, but the relative tradeoff with collagen specificity reduces its effectiveness in quantifying collagen fiber organization. Our work provides insight on both the limitations of the ESEM for tissue imaging, and the potential opportunity to use as a complementary technique when imaging fine features in the non-collagenous regions of tissue samples.

## Introduction

In scanning electron microscopy (SEM) a raster-scanned beam of focused electrons irradiate a sample, the resulting secondary and backscattered electrons are detected and used to form an image of the sample with nanometer resolution^1,2^. The high spatial resolution and fast imaging speed (~1000 megapixels per second) has permitted obtaining structural information and precise measurements of the samples nanostructure in a broad range of applications from material science to biology^1^. Nonetheless, the sample needs to be placed in a vacuum chamber in order to prevent air molecules from scattering the electrons reaching the sample. Hence, the standard procedure for sample preparation usually involves initial dehydration followed by coating with an electrically conductive material, typically gold, which makes it challenging to image biological samples in their natural state.

To mitigate this issue, the environmental-SEM (ESEM) was invented to allow gas inside the sample chamber, through the use of multiple pressure-limiting apertures, while permitting the electron-beam (e-beam) to remain under high vacuum^3,4^. Images are generated by detecting cascaded secondary electrons, which are formed by the collision of secondary electrons ejected by the sample with gas molecules resulting in ionization. The pressure and temperature are both adjustable which result in a desired chamber humidity, thereby prohibiting sample drying. Also, the charging effect, which usually occurs on nonconductive samples, is reduced by the positive ions created by the gas ionization process. Thus, ESEM becomes suitable for imaging fully hydrated and uncoated biological samples. By taking advantage of these features, multiple ESEM studies have reported on the morphological features of mammalian cells^5^, bone^6^, retina^7^ and embryo^4^. ESEM imaging has also been applied to classify different cell and tissue types in histological paraffin sections of rat tongue and has been found to be in agreement with light microscopy analysis, with the added advantage of providing higher resolution^8^. In addition, ESEM imaging has been able to evaluate microstructural damage on potatoes and researchers have suggested the least destructive sample preparation method^9^.

In general, researchers have found that ESEM produces less e-beam induced artifacts on samples, such as shrinkage and cracking, compared to SEM^5^. Some early attempts to understand the effects of the ESEM on biological function looked at continued plant growth^10^ and motion of ants^3^ post e-beam exposure. Other studies have assessed the morphological changes in specimens resulting from ESEM imaging. In one case, the mean volume of yeast cells was shown to shrink from oval-shaped to a flattened pattern^5^. In another example, e-beam exposure was shown to melt down the wax surface layer in plants^11^. Notwithstanding the significance of these studies, damage assessment from ESEM has been mostly qualitative, with little work being done at all on the effect of e-beam exposure on biological tissues.

In this study we apply quantitative second-harmonic generation (SHG) microscopy to investigate the effects of ESEM on collagen fibers in biological tissues, which undergo various sample preparation conditions. SHG is a second-order, nonlinear optical process whereby two impinging photons of identical frequency simultaneously excite a non-centrosymmetric sample, resulting in the emission of a single photon at twice the frequency of the two excitation photons^12,13^. As a biological imaging modality, SHG has high specificity to collagen type I and provides sub-micron 3D resolution^14^. We have previously demonstrated that SHG imaging combined with spatial Fourier analysis (referred to as FT-SHG) provides a simple yet powerful approach to quantifying the collagen architecture. Indeed, this form of quantitative SHG has been applied to the collagenous environments of various samples including tendon^15^, bone^16^, breast tissue^17^ and cervix^18^. Herein, quantitative SHG imaging is used as a tool to compare nearly identical areas before and after e-beam exposure from an ESEM. In addition, selected quantitative parameters are extracted from the SHG images, which reveal the changes in unstained porcine tendon structure caused by ESEM, namely, enhanced cross-linking and structural damage. We also show that the higher spatial resolution afforded by ESEM, compared to SHG imaging, permits visualization of fine structural details, but the relatively lower contrast makes it difficult to estimate individual collagen fiber orientation.

## Results and Discussion

### SHG imaging acquisition and parameter measurements

Near identical areas of porcine tendon sample are imaged using SHG microscopy before and after ESEM imaging (low-vacuum and wet mode). Clear fiducials such as a corner or edge of fiber bundles assist with finding similar regions. Wet-mode ESEM preserves the relative humidity up to 100% in the chamber, thereby preventing evaporation from occurring during imaging. Low-vacuum mode is intermediate between high-vacuum (conventional SEM) and wet mode. Unlike high-vacuum, low-vacuum mode does not require any metal coating on the sample and allows air inside the chamber at a pressure up to 1.0 Torr while imaging. The settings used on the ESEM are referenced from literature^4,5,7,8^ and not aimed to destroy the sample. As a reference, we prepared a control sample where the SHG image pair (before and after e-beam exposure) is obtained without any ESEM imaging. In this case, the sample is left undisturbed for approximately the same amount of time as the experiments carried out with ESEM imaging. In addition, to analyze the effects of sample preparation, fresh and fixed samples are arranged and preserved either frozen or in different stages of dehydration which includes air-dried, dehydrated and critical-point dried. These samples are all imaged under low-vacuum mode, while wet samples with no particular treatment are imaged for wet-mode ESEM. Due to the optical sectioning capabilities of SHG microscopy, all SHG images are obtained as 3D stacks. The before and after SHG images are compared by measuring selected quantitative parameters including density *I*_*d*_, peak spectral intensity *I*_*s*_ and ratio *r*. Details of the experiment is listed in the *Materials and Methods* section.

**Figure 1:**
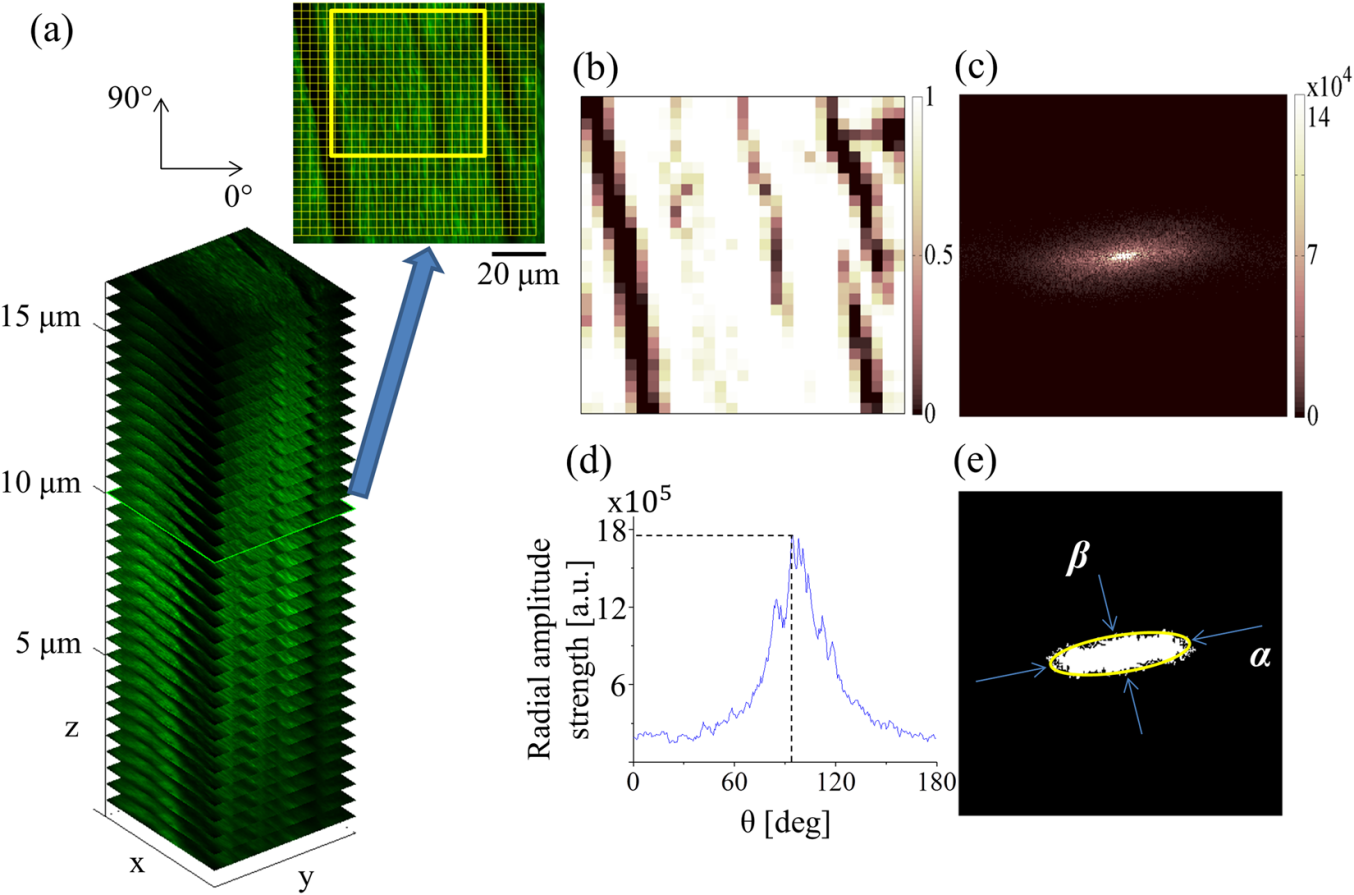
Process of extracting quantitative information from SHG images. (**a**) The obtained 3D SHG-image stack and an image (100 × 100 μm) from a single plane. The yellow box indicates the selected region where peak spectral intensity *I*_*s*_ and ratio *r* is calculated. On the grid, a cell (16 × 16 pixels) shows the scale where the SHG area fraction is calculated. (**b**) A color map of the SHG area fraction of each cell. (**c**) The spatial-frequency domain image (magnitude spectrum) of the yellow boxed region. (**d**) Plot of the spatial-frequencies strength versus orientation angle and peak spectral intensity *I*_*s*_. (**e**) The binarized Fourier domain and ratio *r* obtained by measuring the ratio of the long axis *α* to short axis *β*.

A representative SHG image stack is shown in Fig. 1(a), with each slice in the stack comprising a grid of cells (16 × 16 pixels) and a field-of-view of 100 × 100 μm. The images are analyzed using a customized MATLAB (R2013a, Mathworks) code. In each cell, the area fraction referring to the number of pixels above the noise level divided by the total number of pixels is counted^19^. Thus, each cell will be given an area fraction value ranging from 0 to 1, where 0 indicates the entire cell has no SHG signal and 1 means all the pixels belonging to the cell contains an SHG signal. The selected yellow boxed area is chosen where the majority of fibers are in a uniform orientation, i.e., aligned along the same direction, and there are less dark areas. The color map in Fig. 1(b) illustrates each area fraction value, and the average value from the entire gridded region represents the parameter defined as density *I*_*d*_ of the image. The other two parameters, peak spectral intensity *I*_*s*_ and ratio *r*^20,21^, are obtained in the spatial-frequency domain. In particular, the 2D Fourier transform is applied on the selected region [Fig. 1(c)] and radial amplitude strength versus angle is plotted [Fig. 1(d)] by radially integrating the intensity profile from 0° to 180° along each angle. The maximum value is defined as peak spectral intensity *I*_*s*_. On the spatial-frequency domain image [Fig. 1(c)], low intensity pixels are removed followed by intensity normalization and conversion to a binary image [Fig. 1(e)]. This image is fitted to an ellipse and the ratio *r*, which is the length of the long axis *α* to the short axis *β*, is calculated. All three parameters are measured throughout a z-stack and the average value is acquired.

### E-beam effects observed in SHG images

**Figure 2:**
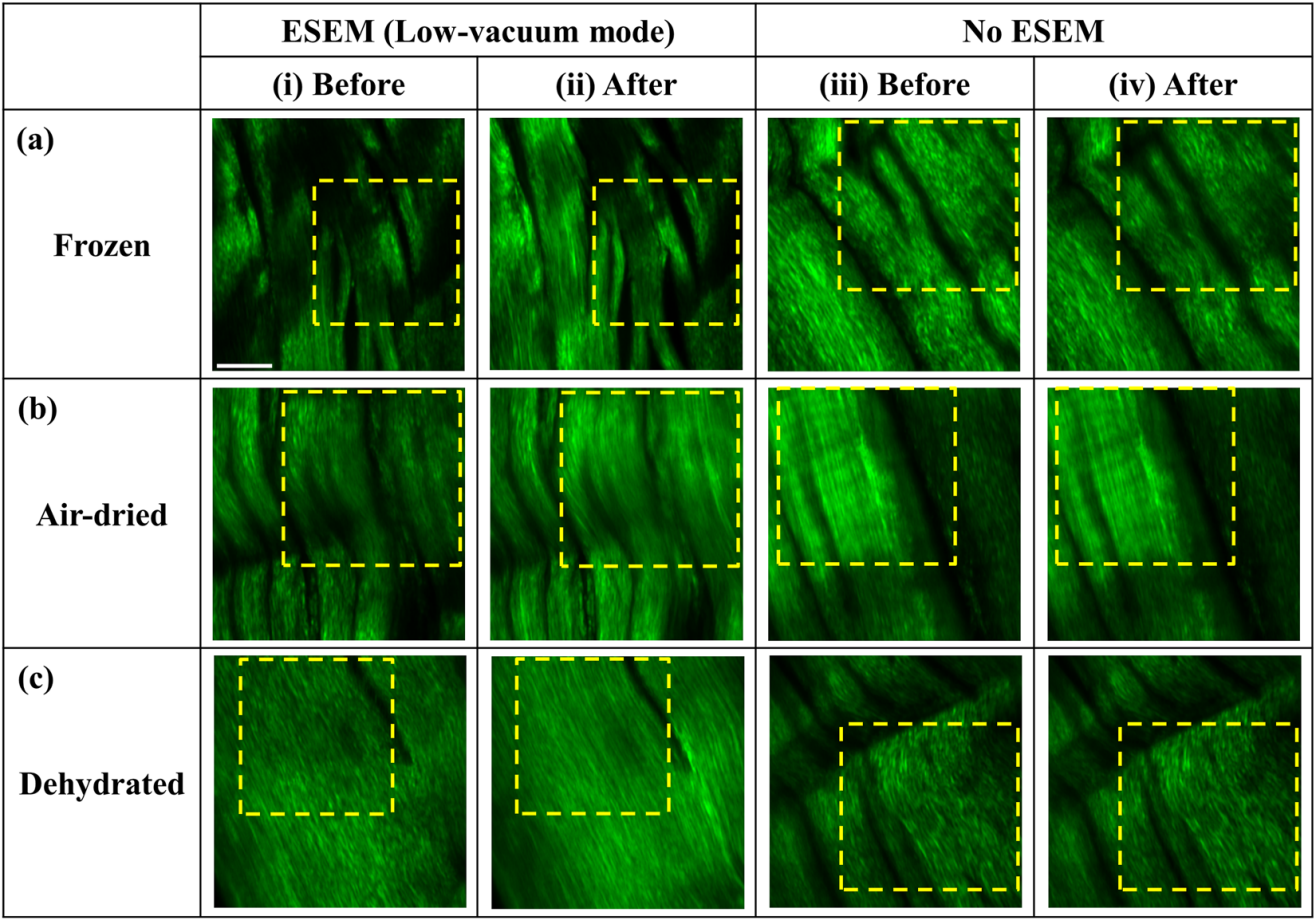
Comparison of the SHG images of tendon samples before and after ESEM exposure in low-vacuum mode. (**a**) Frozen samples. (**b**) Air-dried samples. (**c**) Dehydrated samples. For ESEM exposure (i) and (ii) correspond to before and after exposure, respectively. The no ESEM SHG images (iii) and (iv) correspond to images taken 1 day apart. All compared images are acquired from the identical spatial region. The yellow dotted line areas are selected regions for measuring *I*_*s*_ and *r*. Electron voltage for low-vacuum mode ESEM is 5 kV. Scale bar is 20 μm and applies to all images.

Figure 2 shows SHG images of the collagen fibers in fresh tendon samples that are frozen [Fig. 2(a)], air-dried [Fig. 2(b)], and dehydrated [Fig. 2(c)] before and after ESEM exposure in low-vacuum mode, as displayed in columns (i) and (ii), respectively. Based on the images taken before ESEM exposure, we observe under the ESEM column of Fig. 2(a–c) (i) punctate fiber structures especially in the selected regions of interests shown by the yellow dotted line in each image. These dotted squares are the selected regions chosen for measuring *I*_*s*_ and *r*. In Fig. 2(a–c) (ii), after ESEM exposure, fibers generally appear to have a more continuous fiber structure and higher SHG intensity. The punctuate feature in SHG images is an indicator of less cross-linked fibers and has previously been observed in immature collagen fibers^22,23^ and fibers in late gestation stages^24^. This implies that the e-beam in ESEM potentially promotes cross-linking between collagen fibers. Enhancing cross-linking by e-beam irradiation is a well-established method in manufacturing to improve the mechanical properties and chemical stability of polymers^25,26^. This cross-linking is generated as the irradiation forms polymer molecules to have an unpaired electron which leads to a covalent bond between free radicals^27,28^. Also, induced cross-linking on collagen caused by gamma and e-beam irradiation has been examined^29,30^. However these studies analyze e-beam exposure on different samples whereas we demonstrate the comparison of collagen fibers before and after the ESEM imaging on identical areas using SHG microscopy. Thus our approach is the first to our knowledge to directly observe a potential causal relationship between e-beam irradiation and collagen fiber organization. The gaps between the fiber bundles also appear to widen [Fig. 2(a,i,ii)] due to shrinkage effects^31,32^. Both the induced cross-linking and shrinkage constantly appear throughout the z-stack shown in Supplementary Video S1, thereby indicating that the observed change in collagen fibers is not an artifact of focusing on different planes in the sample. As expected, without any ESEM imaging, we do not observe any difference between initial SHG images taken (‘before’) and those taken 1 day later (‘after’) as shown on the No ESEM row in Fig. 2(a–c). Supplementary Video S2 shows minimal differences between the ‘before’ and ‘after’ SHG image pair with no ESEM imaging in between and the associated Fourier analysis throughout the z-stack. Thus, the lapse of time, at the scale of a single day, does not play a role on the cross-linking effect. We further analyze the effects of the e-beam exposure by demonstrating the extreme case of e-beam-induced damage on the sample using high voltage and magnification [Supplementary Fig. S1].

**Figure 3:**
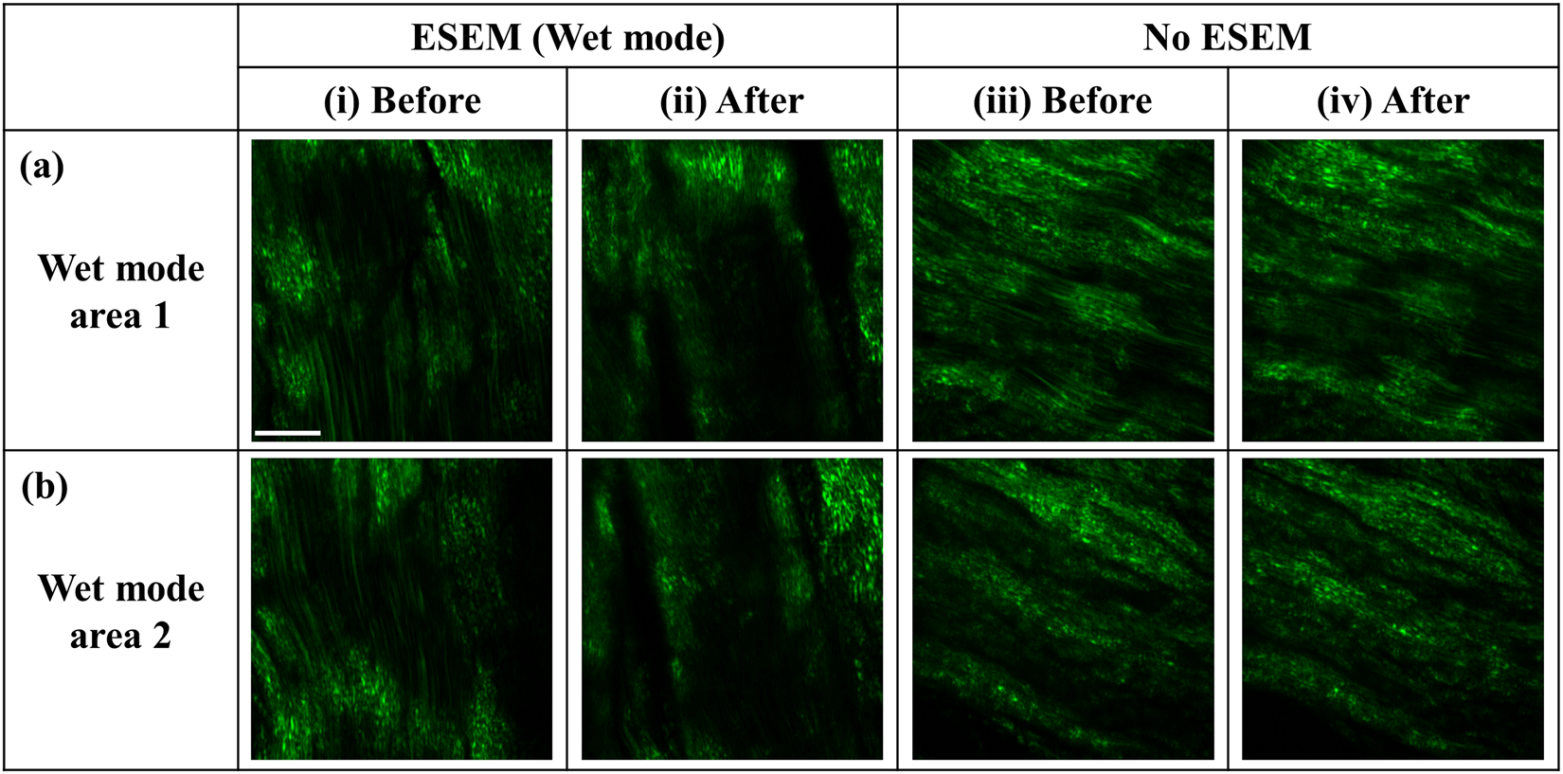
Comparison of the SHG images of tendon samples before and after ESEM exposure in wet mode. (**a**), (**b**) Two fibrous areas chosen close to a reference region where there is little to no irradiation of the e-beam. (i) and (ii) correspond to images obtained before and after, respectively, using ESEM imaging in wet mode. (iii) and (iv) correspond to images taken with the same time interval but without ESEM exposure. The accelerating electron voltage used for wet-mode ESEM is 20 kV. Scale bar is 20 μm and applies to all images.

Figure 3 illustrates the comparison of SHG images of the collagen fibers before and after ESEM exposure in wet mode, as displayed in columns (i) and (ii), respectively. The overall structure of the areas exposed to the e-beam at 1600x or higher magnification radically evolves. As a result, co-registration of the identical area, imaged before e-beam exposure, becomes extremely difficult. Consequently, we choose a wide fibrous area located close to a clear reference region where there is little to no irradiation by the e-beam and obtain two images [Fig. 3(a,b)] on the selected fibrous area. This reference region assists with locating the area after ESEM imaging, and helps to verify that the SHG images observed after e-beam exposure are taken less than 200 μm apart compared to those taken before ESEM imaging. As shown in Fig. 3(a,b,i and ii), the overall structure alters and more dark areas form. This structure variation could be caused by the high voltage (20 kV) e-beam used in the wet mode to compensate for the low contrast in ESEM imaging. The time interval between the before and after e-beam exposure of SHG images is approximately 3 hours. Conversely, the SHG image pair obtained with the same time interval, but without ESEM imaging, appear to nearly be identical [Fig. 3(a,b,iii and iv)]. This indicates that the drying that occurs in the aforementioned time interval has a negligible effect on any observable collagen structure. The effect of e-beam exposure in wet mode is also demonstrated by bright-field microscopy in Supplementary Fig. S2 (see Supplementary Notes for details).

### Quantitative analysis of e-beam exposure on the SHG images

**Figure 4:**
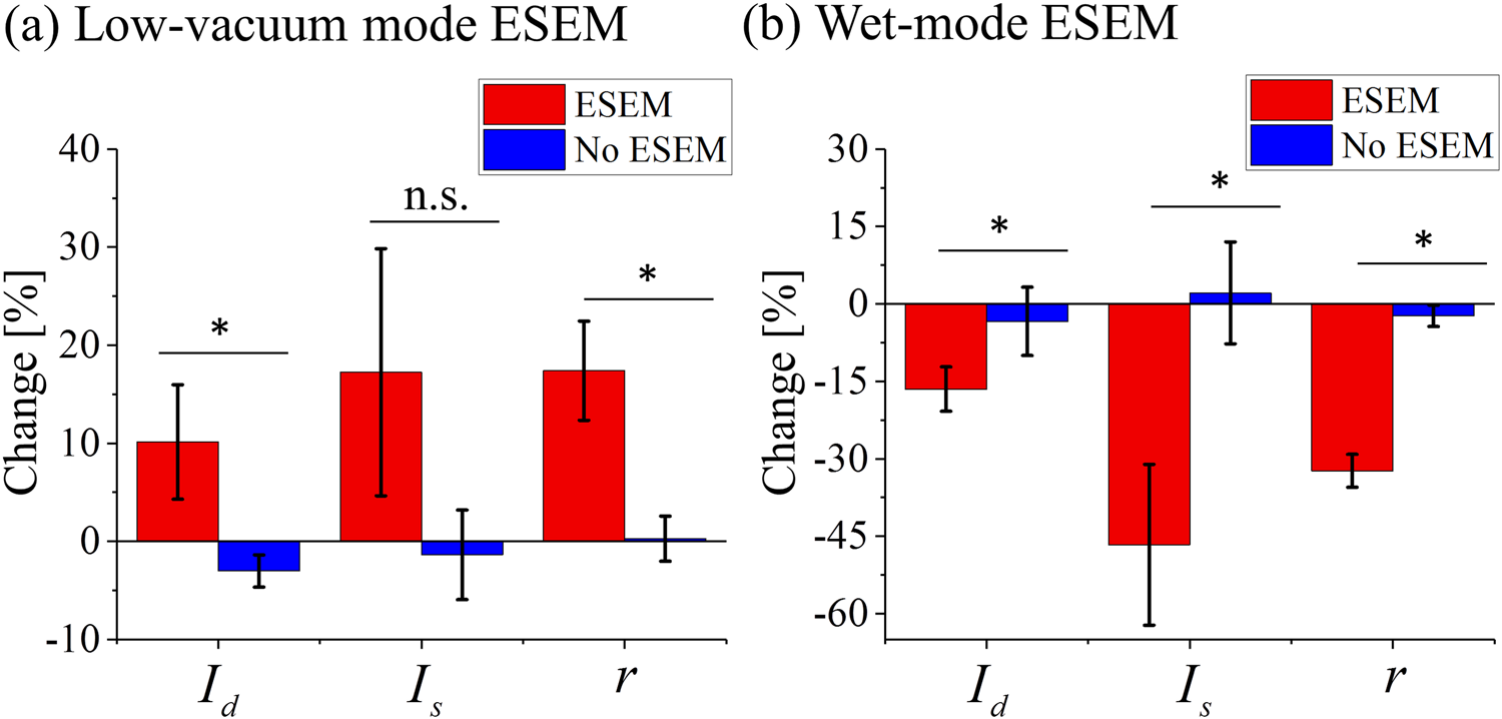
Parameter changes measured from SHG images as a function of ESEM mode. The average changes of the parameters (density *I*_*d*_, peak spectral intensity *I*_*s*_ and ratio *r*) resulting from (**a**) low-vacuum mode ESEM imaging (red; n = 3) and with no ESEM imaging (blue; n = 3). The value is averaged for the frozen, air-dried and dehydrated samples. Change caused by (**b**) the wet-mode ESEM imaging (red; n = 3) and with no ESEM imaging (blue; n = 4). The data shown are mean ± standard deviation; n.s., non significant; *p < 0.05.

The parameters explained in Fig. 1 (*I*_*d*_, *I*_*s*_ and *r)* are measured on the SHG image pair (before and after e-beam irradiation) and the amount of change in these parameters is plotted in Fig. 4. The calculated parameters on each SHG image are averaged for the entire stack and repeated on frozen, air-dried and dehydrated samples. The mean values obtained from the three types of samples are plotted in Fig. 4(a). For the low-vacuum mode [Fig. 4(a)] ESEM group, *I*_*d*_ (10.14 ± 5.83%), *I*_*s*_ (17.25 ± 12.60%) and *r* (17.41 ± 5.06%) all increase more than 10%, whereas fibers analyzed without ESEM imaging (No ESEM group) have a change smaller than 4%: −3.01 ± 1.64% for *I*_*d*_, −1.36 ± 4.57% for *I*_*s*_ and 0.28 ± 2.30% for *r*. The difference between the ESEM and No ESEM groups for *I*_*d*_ (*p* = 0.020) and *r* (*p* = 0.006) are shown to be statistically significant (*p* < 0.05). With respect to *I*_*s*_ a *p* value slightly greater than 0.05 (*p* = 0.074) was obtained. The reason for the increase in the parameters after ESEM exposure is likely because of the induced cross-linked fibers. Cross-linked fibers have less graininess in SHG images, resulting in a stronger intensity along the preferred orientation in the spatial-frequency domain image. This results in a larger peak in the magnitude spectrum [Fig. 1(d)] and also increases the length of the major axis in the binarized Fourier domain [Fig. 1(e)]. In addition, strongly cross-linked fibers appear to have a higher intensity in the SHG image compared to less cross-linked fibers. In other words, the induced cross-linking effect is the cause of the change on all three parameters. The density value does not increase as much as the other two parameters because of the widening of the gaps between fiber bundles. However, we analyze targeted areas where fibers occupy most of the image and therefore the density value after e-beam exposure increases.

Figure 4(b) shows the change of average-parameter values caused by wet-mode ESEM. The average values are acquired from two broad fibrous areas, each including two SHG images. This is because, as previously mentioned, the e-beam damage of the sample makes it difficult to compare identical areas and thus, broad fibrous areas are chosen. All parameters for the fibers that are exposed to the wet-mode ESEM decrease more than 16% (*I*_*d*_ = −16.49 ± 4.30%, *I*_*s*_ = −46.65 ± 15.59%, *r = *−32.31 ± 3.18%), as opposed to the No ESEM group parameters (*I*_*d*_ = −3.36 ± 6.62%, *I*_*s*_ = 2.15 ± 9.87%, *r = *−2.30 ± 2.07%) that have minimal change less than 4% [Fig. 4(b)]. The difference between ESEM and No ESEM groups for all parameters are shown to be statistically significant (*p* < 0.05): *p* = 0.032 for *I*_*d*_, *p* = 0.004 for *I*_*s*_ and *p* = 0.00002 for *r*. The decrease of the density reflects the increasing dark areas and the other two parameters dropped as a result of the fiber orientation and overall structure becoming more diffuse and irregular in the SHG images. The reason for the total structural change observed in Fig. 4(b) could be due to the water molecules in the samples resulting in ionization and chemical breakdown of the sample^33,34^. As a result, hydrated samples become more vulnerable to beam irradiation compared to dried samples. For example, researchers have found the increase of beam damage on hydrated polypropylene^34^. Another reason for the collagen fiber samples being damaged could be the increased accelerating voltage used in wet-mode ESEM to enhance the contrast. Overall, *I*_*s*_ and *r* could be used as a measure of the cross-linking occurring in fibers and also to detect damage happening on the sample. The error bars indicate the standard deviation. The fresh critical-point dried samples and all the fixed samples do not show any sign of cross-linking or change in structure after e-beam exposure, which complies with previous studies conducted on neuronal cells demonstrating the stability of the fixed samples during ESEM imaging^35^. The SHG images and quantitative analysis for fixed samples are in Supplementary Figs S3 and S4.

### Advantages and limitations of ESEM on collagen fiber imaging

**Figure 5:**
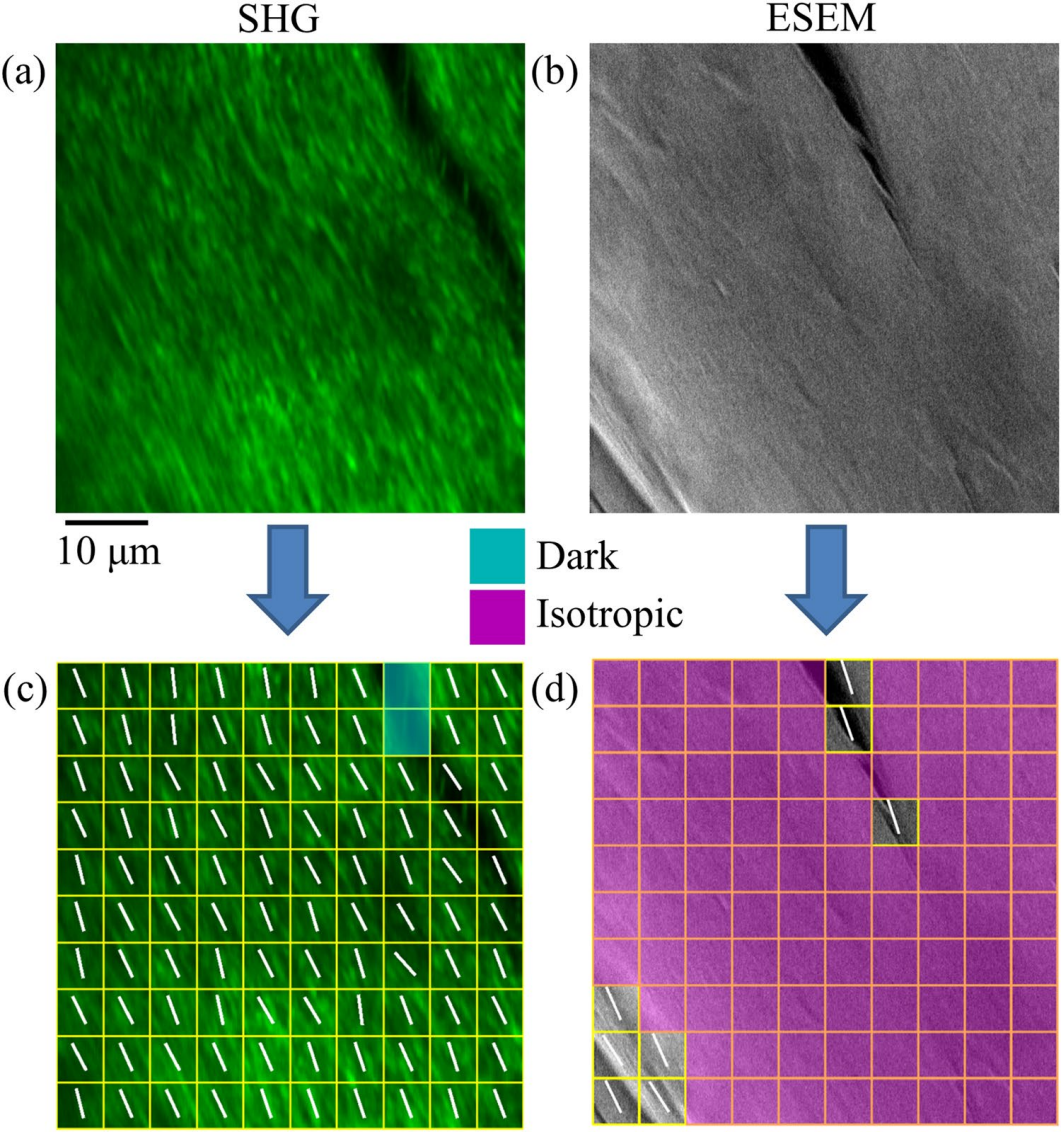
Comparison of the computed preferred collagen fiber orientation derived from SHG and ESEM images. Representative (**a**) SHG and (**b**) ESEM images of collagen fibers, and (**c**), (**d**) their respective computed fiber orientations. Cyan highlights regions with little or no SHG signal, while purple highlights regions with spatially isotropic orientation of fibers. Refer to reference^36–38^ for details. Scale bar applies to all images.

Figure 5 shows the result of preferred fiber orientation analysis using the image gradient method^36–38^ derived from SHG and ESEM collagen fibers images. This analysis categorizes the predetermined gridded areas into anisotropic, isotropic and dark areas and computes the preferred fiber orientation. Representative SHG and ESEM images are shown in Fig. 5(a) and (b), respectively, and the associated measured fiber orientation results are in Fig. 5(c,d). For the SHG image 98 cells are detected as anisotropic and the measured preferred orientations are along the actual fiber orientation. Two cells in the grid are identified as dark regions due to the low intensity. For the ESEM image only 8 cells in the grid are determined as anisotropic, while the remaining cells are considered isotropic and thus have no preferred orientation. This demonstrates that the intrinsic specificity to fibrillar collagen afforded by SHG microscopy makes it a more suitable tool for identifying individual fiber orientation in comparison to ESEM, where the spatial resolution is higher but with little specificity to collagen^16^.

**Figure 6:**
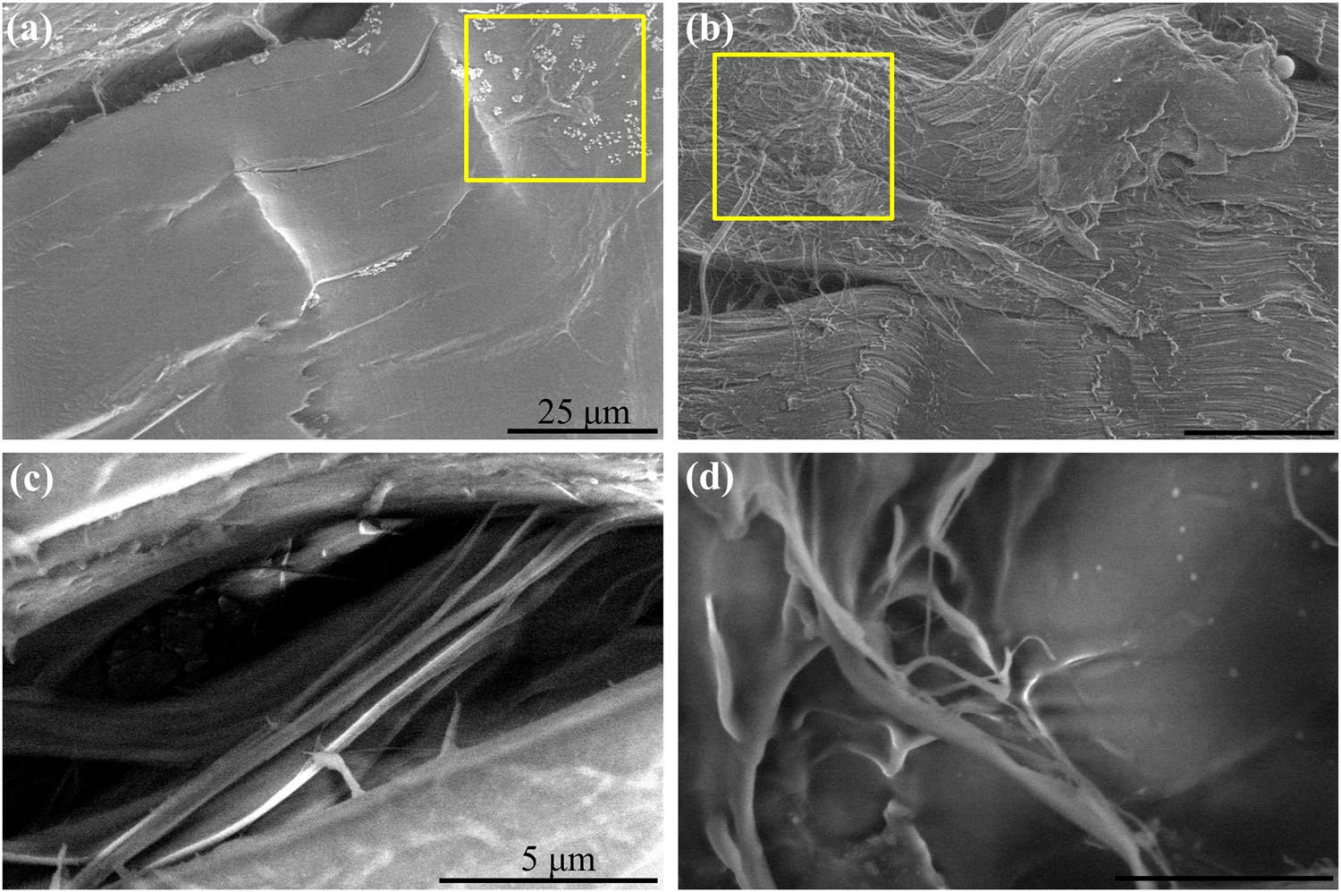
Fine features on collagen fiber samples observed in ESEM imaging. The yellow box illustrates (**a**) the remaining OCT compound used for embedding the sample and (**b**) tangled fibers on top of straight fibers. Scale bar is 25 μm for both images. (**c**), (**d**) Interfascicular connective tissue referring to open spaces between collagen fiber bundles. Scale bar is 5 μm for both images.

In spite of the fiber structure alteration and damage generated by the e-beam, there are unique features which highlight the advantages of ESEM as shown in Fig. 6. The identified yellow boxed areas in Fig. 6(a,b) represent components on the sample surface, which are the remaining optimal cutting temperature (OCT) compound used for embedding the sample, and tangled fibers on top of straight fibers, respectively. ESEM also becomes useful for observing details on the nanometer scale within the sample^39^. Figure 6(c,d) are images of interfascicular connective tissue^40^, which are open spaces between collagen fiber bundles. Interfascicular connective tissues have fibroblasts and numerous blood tissues and researchers have investigated age-related alterations in interfascicular matrix^41^ and their correlation with mechanical properties^42^ and muscle atrophy^43^ in tendon. Overall, the aforementioned features are difficult to image using a SHG microscope due to the lower resolution and strong collagen type I specificity.

## Conclusion

In this paper the effects of ESEM imaging on collagen fibers were analyzed using quantitative SHG microscopy. We showed that for frozen, air-dried and dehydrated samples, the e-beam for low-vacuum ESEM imaging promotes cross-linking between fibers, while fixed samples remain unaffected. For wet samples imaged by wet-mode ESEM, structure degradation was observed. The change of structure was quantified by measuring parameters derived from the average SHG image intensity and corresponding spatial-frequency analysis. We observed that these parameters increased in low-vacuum mode and decreased in wet mode subsequent to ESEM imaging. Furthermore, we confirmed that while ESEM provides higher spatial resolution than optical microscopy, the specificity to collagen fibers is relatively low. The aforementioned results suggest that to further extend ESEM to *in vitro* applications, thoroughly analyzing its impact on the sample will be critical. However, the technology holds potential as a technique for analyzing the non-collagenous regions of tissues with nanometer resolution.

## Materials and Methods

### Sample preparation

Porcine feet were purchased from a local abattoir and stored in the freezer. Subsequently tendon tissue was dissected after the samples being thawed overnight.

For fresh samples, tendon was embedded in OCT compound and cut into 25-μm thick slices by a cryostat (CM3050, Leica). Slices were mounted on glass slides and either frozen or treated by three different levels of drying procedures. Frozen samples were stored in the freezer and taken out solely for imaging. Air-dried samples were left dried in air for at least 24 hours. Dehydrated samples were damped in incremental levels of ethanol (37%, 67%, 95%, 100% - three times) for 10 minutes each. Critical-point dried samples went through the abovementioned dehydration process followed by critical-point drying (Samdri-PTV-3D, Tousimis) and lastly were embedded in paraffin wax and cut by a microtome (CM3050, Leica).

To fix samples, dissected tendon samples were maintained in paraformaldehyde for 1 hour in vacuum. Air-dried samples followed the same protocol as the fresh samples. The other fixed samples (dehydrated, critical-point dried) were carried out as explained previously and imbedded in paraffin wax as the final step.

Wet samples did not go through any fixation or drying process and were embedded in OCT compound and cut into thin slices. All wet samples slices were cut to a 4 × 4-mm dimension in order to fit on a custom made 6 × 6-mm glass substrate. The samples were kept in a petri dish hydrated by placing buffer solution droplets on the sample. To prevent decomposition, samples were stored in a refrigerator until imaging.

### SHG imaging

A tunable Ti:Sapphire laser (Mai Tai, Spectra-Physics) generating 100-fs duration pulses and centered at 780-nm wavelength illuminates the sample. The 10-mW power beam is then focused on the sample by a 40x, 0.65 numerical aperture (NA) objective lens (PLAN N, Olympus) and the backward SHG emitted by the sample is collected by a 390-nm bandpass filter (FF01-390/18-25, Semrock). Wet samples are placed on top of a standard microscope slide (25 × 75 mm) in order to be secured on the microscope stage. On each sample a targeted fibrous area with clear fiducials such as edges and wide gaps between fiber bundles is chosen for SHG imaging for co-registration. A motorized stage (max5000, Ludl) provides minute translational adjustments of the sample on the stage. All samples but the wet are imaged in 3D stacks of dimension 100 × 100 × 15 μm; the step size along the z axis is 500 nm. The wet samples are imaged in 2D (100 × 100 μm). Details of the optical setup have been described elsewhere^44^.

### ESEM imaging

Fresh and fixed samples are imaged with the low-vacuum mode ESEM (Quanta FEG 450 ESEM, FEI) by placing the microscope slide directly on the stud. The pressure inside the chamber is 0.98 Torr and the accelerating voltage is 5 kV. The distance between the sample and e-beam (working distance) is 9 mm. Initially low magnification is used to identify the targeted area and further increased up to 1600x on the targeted area. For each ESEM image the pixel dwell time is 10 μs; to assure the entire targeted area is exposed to the e-beam, we also probe and image the surrounding area, which takes roughly 15 minutes in total. For wet-mode ESEM, the 6 × 6-mm glass slide is attached to double-sided black tape to not only enhance the contrast but also mount the sample on the stud being placed on the Peltier stage. The target temperature is 4 ° and the pressure is 6.1 Torr, which is selected to generate 100% humidity inside the chamber. A (gaseous) secondary electron detector is used to generate the image. The magnification and working distance is identical with the low-vacuum mode. Due to the low contrast caused by the water molecules inside the chamber, the accelerating voltage is increased to 20 kV.

### Statistical analysis

The data are represented as mean ± standard deviation. Groups are compared with the unpaired two-tailed Student’s t-test for small sample sizes^45^. The significant *p* value is 0.05.

### Data availability

Relevant data that support the results of this study could be found within the paper, Supplementary Information and from the corresponding author upon request.

## Electronic supplementary material

Supplementary Information

Video S1

Video S2
