# Supplementary material for: Quantitative analysis of the effect of environmental-scanning electron microscopy on collagenous tissues
Source: Sci Rep. 2018 May 31;8:8491. doi: 10.1038/s41598-018-26839-x (PMC5981445; doi:10.1038/s41598-018-26839-x)
Supplement: Supplementary file 1 — Supplementary Information [file 41598_2018_26839_MOESM1_ESM.pdf]

Quantitative analysis of the effect of environmental-scanning electron microscopy on collagenous tissues

Woowon Lee[a,b] and Kimani C. Toussaint, Jr.[a,b,c,d,*]

[a]University of Illinois at Urbana-Champaign, Department of Mechanical Science and Engineering, 1206 W Green Street, Urbana, Illinois 61801, United States

[b]University of Illinois at Urbana-Champaign, PROBE Lab, 1206 W Green Street, Urbana, Illinois 61801, United States

[c]University of Illinois at Urbana-Champaign, Affiliate in the Department of Electrical and Computer Engineering, 1406 W Green Street, Urbana, Illinois 61801, United States

[d]University of Illinois at Urbana-Champaign, Affiliate in the Department of Bioengineering, 1270 Digital Computer Laboratory, Urbana, Illinois 61801, United States

[*]**Address all correspondence to:** Kimani C. Toussaint, Jr., Telephone : 217-244-4088, E-mail : ktoussai@illinois.edu

Supplementary Notes

E-beam (low-vacuum mode) induced sample damage. Significant differences are observed for both SHG and ESEM images of the same regions taken before and after e-beam exposure as shown in Figs. S1(a), (d) and Figs. S1(b), (c), respectively. The sample is intentionally left exposed under the e-beam for approximately 1 minute under high voltage (20 kV) and magnification (> 1600x). The pixel dwell time is 10 μs and the operating ESEM mode is low vacuum. The collagen content decreases, creating craters observed in both the ESEM [Fig. S1(c)] and SHG images [Fig. S1(d)]. The surrounding areas also change substantially, namely the gaps between collagen fibers widen; we also note that the SHG intensity in these same regions decrease. These results could be potentially attributed to the highly concentrated e-beam bombardment of the sample, thereby generating bond scission and surface temperature increase[1,2].

E-beam (wet mode) effects demonstrated by bright-field microscopy. We observe the sample microstructure before and after e-beam irradiation under wet-mode ESEM using bright-field microscopy. A commercial microscope (Axio Observer Z1, Zeiss) with a 10x, 0.3 numerical aperture (NA) objective lens is used to obtain 16 images (4 x 4), which are tiled to form a 1500 x 1500-μm image [Fig. S2(a) (i)]. The image includes a selected area for e-beam irradiation shown by red dotted lines in Fig. S2(a). We next zoom in to the targeted area by using a 20x, 0.6 NA objective lens. As a reference, bright-field microscopy experiments are also conducted without any ESEM imaging [Fig. S2(b)] with an interval of 2 hours between images labeled "before" [Fig. S2(b) (i)] and "after" [Fig. S2(b) (ii)], which is similar with the interval used in Fig. S2(a). We observe in the zoomed-in images in Fig. S2(a) a region with regular fibrous structures and the same region without the fibrous features before and after e-beam exposure, respectively. In

Fig. S2(b) the two zoomed-in images do not show a significant difference having similar structural features.

Reference

1. Egerton, R. F., Li, P. & Malac, M. Radiation damage in the TEM and SEM. *Micron* **35,** 399–409 (2004).
2. Boyde, A. & Jones, S. J. Scanning electron microscopy of bone: Instrument, specimen, and issues. *Microsc. Res. Tech.* **33,** 92–120 (1996).

Supplementary Figures

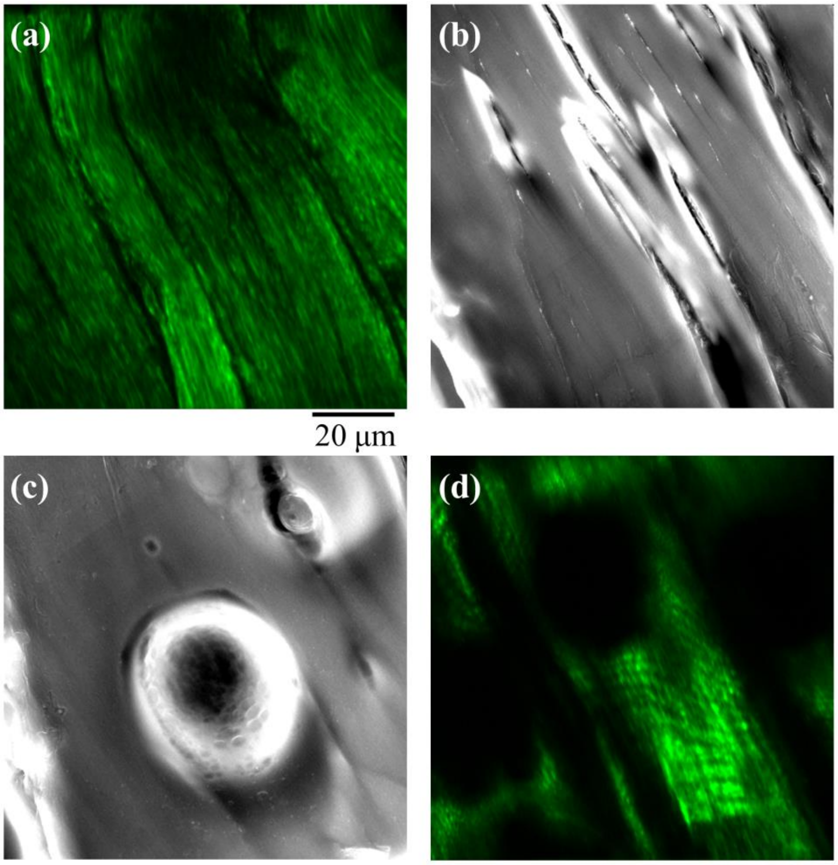

Figure S1. Tissue damage induced by the e-beam. (a) SHG image of a fibrous area without any e-beam exposure. ESEM images (b) before and (c) after e-beam exposure. The setting of the ESEM is 20 kV and 3200x for acceleration voltage and magnification, respectively, and the operating mode is low vacuum. (d) SHG image of a region depicting damage from e-beam exposure. For visualization purposes, the maximum SHG threshold is adjusted. Scale bar applies to all images.

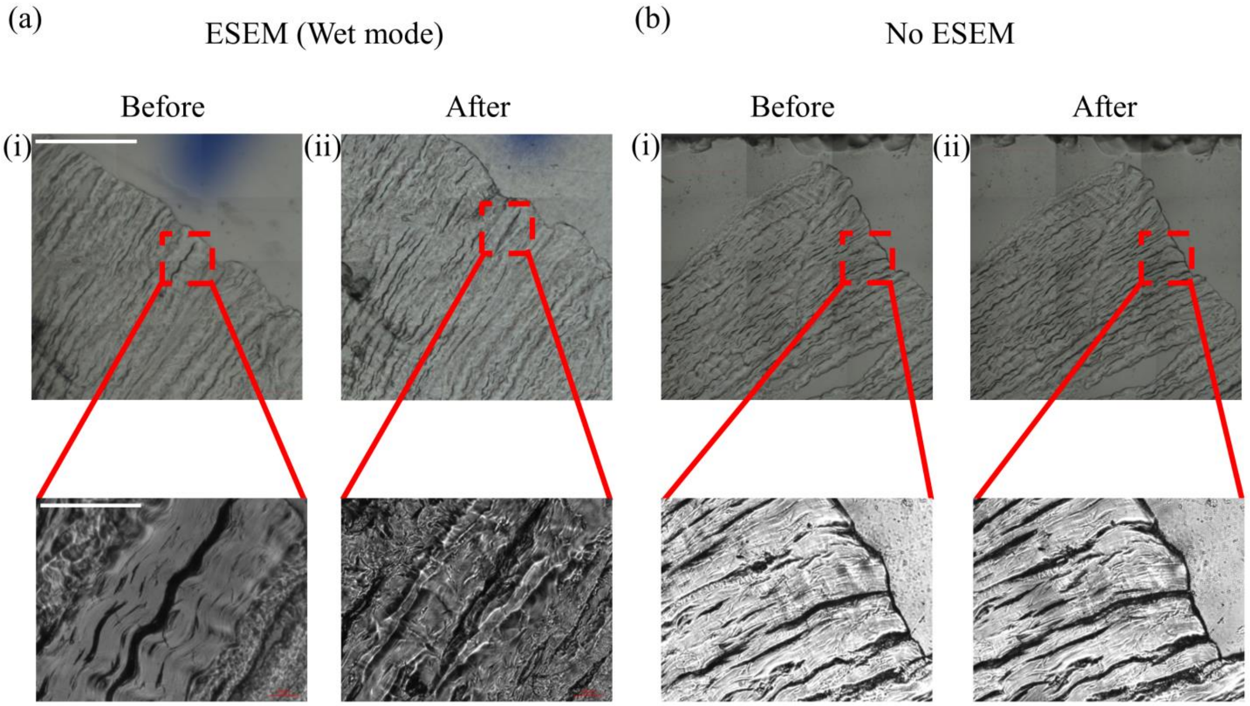

Figure S2. Effects of the e-beam as observed by bright-field microscopy. Two bright-field images are obtained at the time interval of 2 hours (a) with ESEM and (b) without ESEM imaging. A tiled image showing a wide-field view (i) before and (ii) after exposure to the ESEM in wet mode. The associated magnified regions for (a) and (b) are shown in the bottom row. The accelerating electron voltage used for wet-mode ESEM is 20 kV. Scale bar is 600 μm for the tiled images and 150 μm for the magnified images.

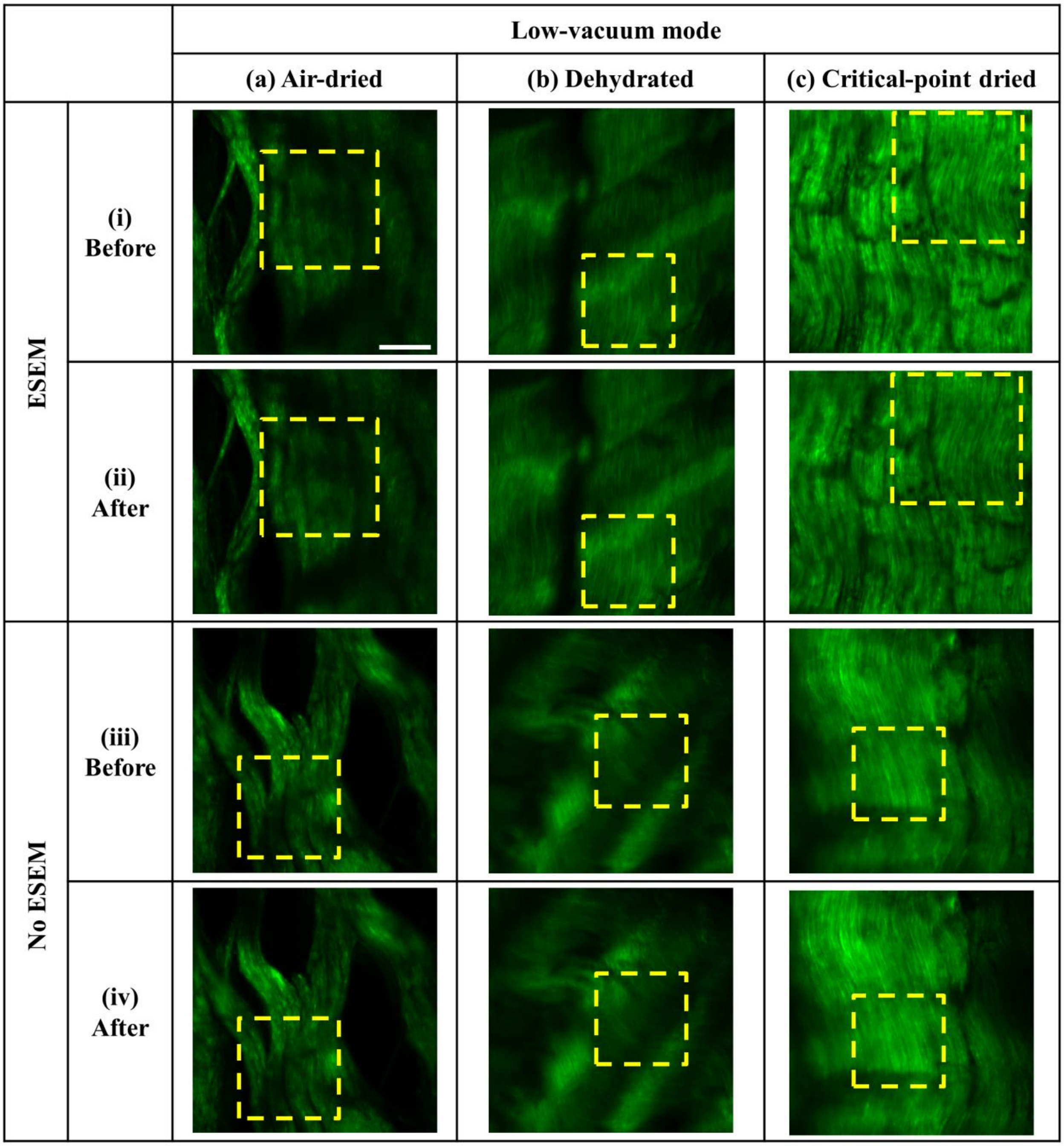

Figure S3. SHG image pairs of fixed tendon samples before and after ESEM imaging in low-vacuum mode. (a) Air-dried samples. (b) Dehydrated samples. (c) Critical-point dried samples. For the ESEM row, (i) and (ii) correspond to before and after e-beam exposure on the identical spatial region, respectively. The No ESEM row SHG images (iii) and (iv) correspond to images taken 1 day apart. The yellow dotted line areas are selected regions for measuring I_s and r. Electron voltage used for ESEM imaging is 5 kV. Scale bar is 20 μm and applies to all images.

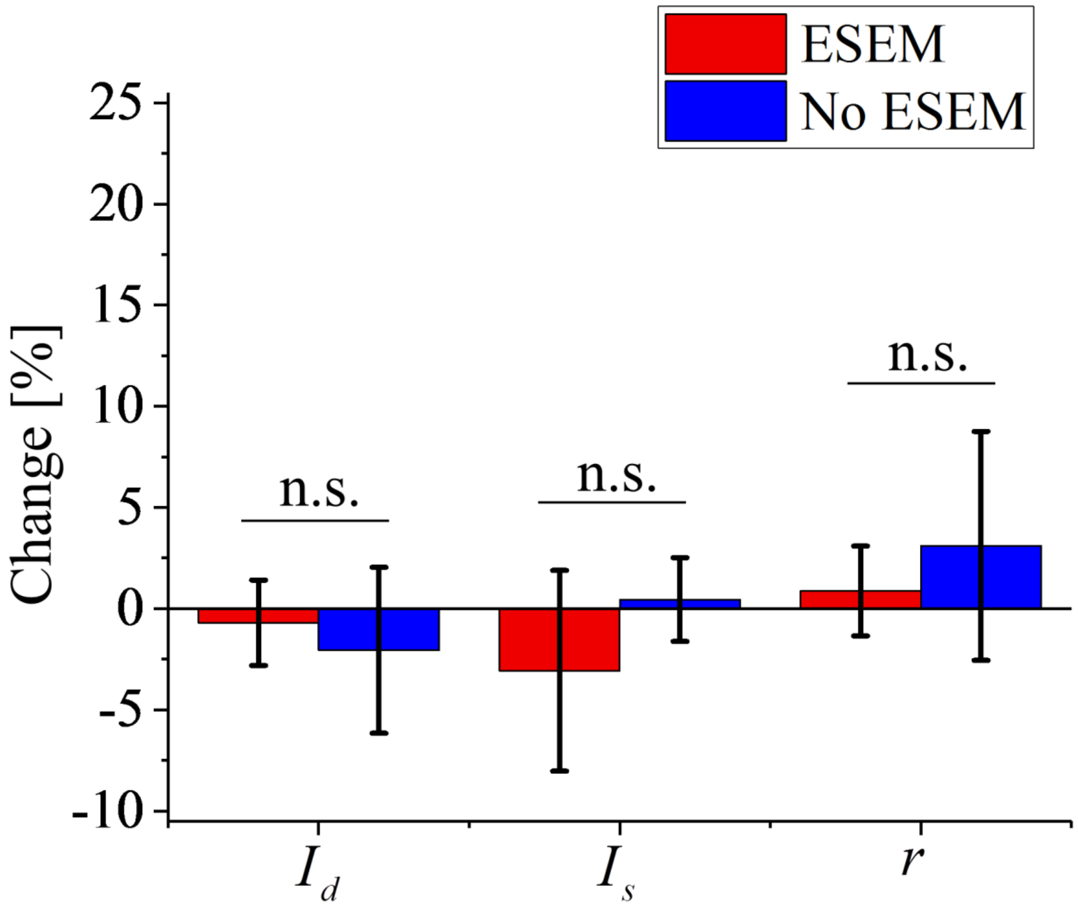

Figure S4. Parameter changes measured from SHG images of fixed samples. The average changes of the parameters (density I_d, peak spectral intensity I_s and ratio r) resulting from low-vacuum mode ESEM imaging (red; n=3) and with no ESEM imaging (blue; n=3). The value is averaged for the air-dried, dehydrated and critical-point dried samples. The data shown are mean ± standard deviation; n.s., non significant.

Supplementary Videos (files attached/available online)

Video S1. Movie of SHG images illustrating the effects of e-beam exposure along the z-stack. The movie shows the obtained SHG images and their corresponding fast-Fourier transform (FFT) analysis. The samples are air-dried and the dimension of the SHG image is 60 x 60 µm.

Video S2. Movie of SHG images obtained along the z-stack on two different days. The movie includes the SHG images and their corresponding FFT analysis. The samples are air-dried and the dimension of the SHG image is 60 x 60 µm.
